# Research status and development trends of omics in neuroblastoma a bibliometric and visualization analysis

**DOI:** 10.3389/fonc.2024.1383805

**Published:** 2024-10-10

**Authors:** Mengliang Han, Huizhong Niu, Fei Duan, Zhaolong Wang, Zhiguang Zhang, Hui Ren

**Affiliations:** First Department of General Surgery, Hebei Children’s Hospital, Shijiazhuang, Hebei, China

**Keywords:** bibliometrics, omics, neuroblastoma, Citespace, VOSviewer

## Abstract

**Background:**

Neuroblastoma (NB), a prevalent extracranial solid tumor in children, stems from the neural crest. Omics technologies are extensively employed in NB, and We analyzed published articles on NB omics to understand the research trends and hot topics in NB omics.

**Method:**

We collected all articles related to NB omics published from 2005 to 2023 from the Web of Science Core Collection database. Subsequently, we conducted analyses using VOSviewer, CiteSpace, Bibliometrix, and the Bibliometric online analysis platform (https://bibliometric.com/ **).**

**Results:**

We included a total of 514 articles in our analysis. The increasing number of publications in this field since 2020 indicates growing attention to NB omics, gradually entering a mature development stage. These articles span 50 countries and 1,000 institutions, involving 3,669 authors and 292 journals. The United States has the highest publication output and collaboration with other countries, with Germany being the most frequent collaborator. Capital Medical University and the German Cancer Research Center are the institutions with the highest publication count. The Journal of Proteome Research and the Journal of Biological Chemistry are the most prolific journal and most co-cited journal, respectively. Wang, W, and Maris, JM are the scholars with the highest publication count and co-citations in this field. “Neuroblastoma” and “Expression” are the most frequent keywords, while “classification,” “Metabolism,” “Cancer,” and “Diagnosis” are recent key terms. The article titled “Neuroblastoma” by John M. Maris is the most cited reference in this analysis.

**Conclusion:**

The continuous growth in NB omics research underscores its increasing significance in the scientific community. Omics technologies have facilitated the identification of potential biomarkers, advancements in personalized medicine, and the development of novel therapeutic strategies. Despite these advancements, the field faces significant challenges, including tumor heterogeneity, data standardization issues, and the translation of research findings into clinical practice.

## Introduction

Neuroblastoma (NB), the most prevalent extracranial solid tumor in children, is a malignant tumor arising from neural crest cells (1). NB originates from neural crest cells found in the sympathetic nervous system, typically situated in either the adrenal medulla or paravertebral ganglia. Consequently, it presents as tumor formations in regions such as the neck, chest, abdomen, or pelvic area (2). Clinical presentations vary from asymptomatic masses to severe conditions resulting from local invasion and widespread disease dissemination (3, 4). The incidence rate stands at 10.2 cases per million children below the age of 15, with the majority (90%) occurring in those under 10 years old. These cases make up 15% of pediatric tumor-related fatalities (5, 6).

Omics refers to the study of a certain molecular group, mainly including genomics, transcriptomics, proteomics, metabolomics, imaging omics, and metabolomics (7). Genomics is a scientific field that studies the structure, function, and heredity of the entire genome of an organism. It involves a systematic investigation of the overall genetic information of all genes in an organism, aiming to understand the composition, structure, function, and interactions among genes within the genome (8). Transcriptomics is the comprehensive study of the entire collection of messenger RNA (mRNA) within the cells of living organisms. Its goal is to understand the patterns of gene expression, specifically which genes are transcribed into mRNA under specific conditions and their relative levels (9). Proteomics investigates the composition, structure, function, and interactions of all proteins within a biological organism. The objective is to comprehensively understand the overall expression of proteins in the organism and how these proteins contribute to the structure, function, and regulation of cells (10). Metabolomics is the study of the composition, structure, and changes of all metabolites generated during the metabolic processes in organisms, including amino acids, lipids, sugars, and other metabolic products. The goal is to offer a thorough comprehension of the metabolic features exhibited by organisms in diverse physiological states, environmental conditions, or disease states (11). Radiomics is an interdisciplinary approach that integrates various disciplines such as medical imaging, computer science, and statistics. It involves the quantitative analysis and mining of medical imaging data with the aim of revealing imaging features associated with the occurrence, development, treatment, and prognosis of diseases (12). Omics technologies provide comprehensive insights into the molecular mechanisms, potential biomarkers, and therapeutic targets of NB, ultimately contributing to the diagnosis, treatment, and prognostic strategies for NB.

Bibliometrics, an emerging field, reveals hotspots and trends in specific research areas, contributing to a deeper understanding of the knowledge structure within the academic domain (13).

We employ bibliometrics as a research tool to conduct visual analysis on NB omics in terms of countries, institutions, journals, authors, references, keywords, etc. This approach offers distinctive insights into the present state and research trends of NB omics, enhancing its research by strengthening its analysis.

## Methods

### Data collection

**Figure 1:**
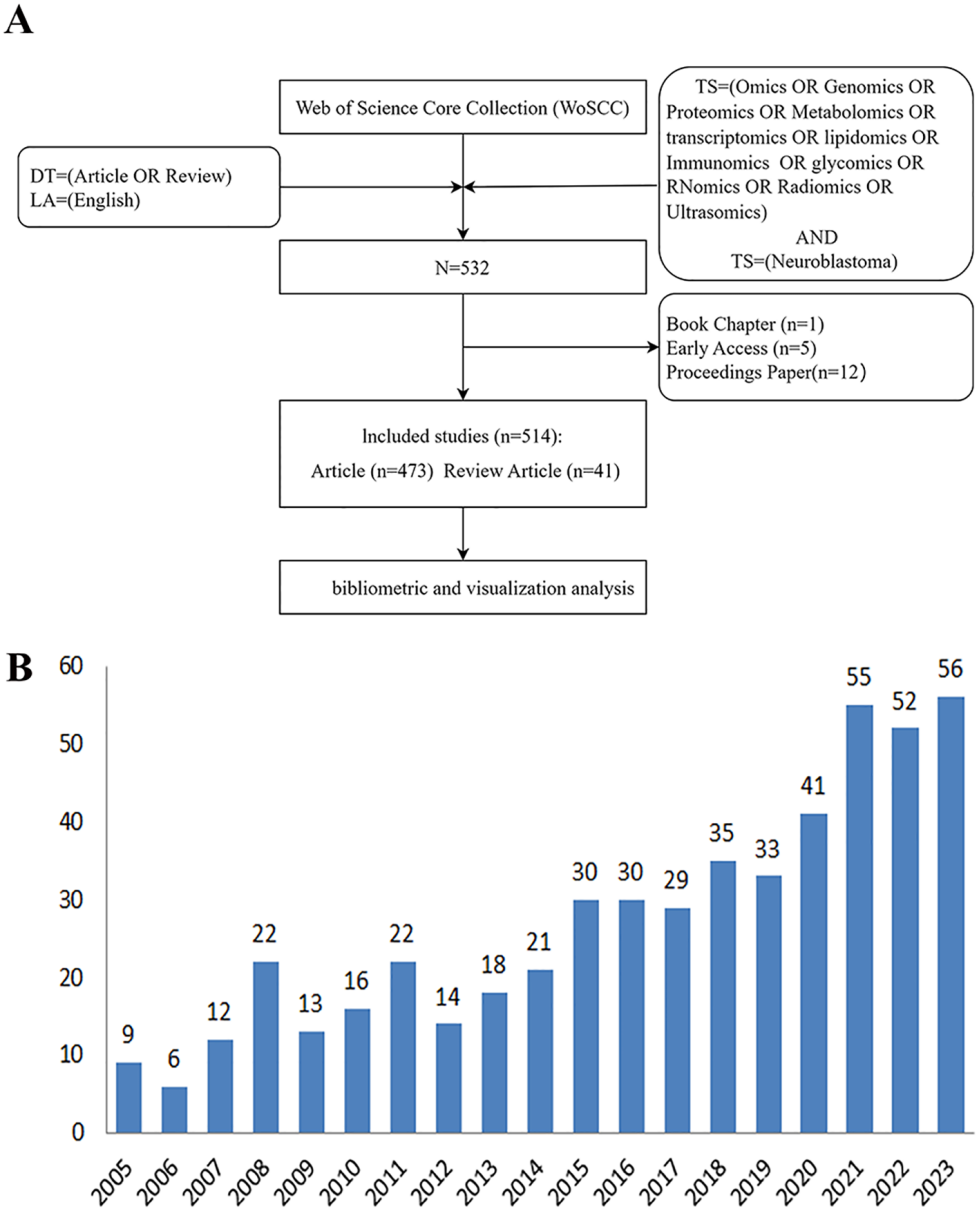
**(A)** The flowchart of publications screening. **(B)** Annual number of publications on omics in NB.

We systematically searched the Web of Science Core Collection (WoSCC) database using the following expressions: TS=(Omics OR Genomics OR Proteomics OR Metabolomics OR transcriptomics OR lipidomics OR Immunomics OR glycomics OR RNomics OR Radiomics OR Ultrasomics) AND TS=(Neuroblastoma) AND The search was limited to articles or reviews, and publications were restricted to English. The search results were obtained in “Full Record and Cited References” and “Plain Text” formats, which were then saved as “Download.txt” files. To ensure reliability, two independent reviewers conducted the literature search, with a third reviewer reviewing the results. All data were retrieved from WoSCC on Jan 17, 2024, to prevent bias from continuous updates. The retrieval strategy process is illustrated in **Figure 1A**.

### Data analysis

We utilized VOSviewer (version 1.6.18), CiteSpace (version 6.2.R7), and Bibliometrix (version 4.1.3) (https://www.bibliometrix.org), along with the Bibliometric Online Analysis Platform (https://bibliometric.com/), to analyze the data in the literature (14).

VOSviewer, developed by the Leiden University Centre for Science and Technology Studies, is utilized for visualizing and analyzing literature networks. In this study, we employed it for visual analysis of institutions, journals, co-cited journals, authors, co-cited authors, keywords, etc. (15). The VOSviewer graph displays cluster types, density colors, or overlays. In the clustering graph, nodes reflect co-occurrence frequencies; links represent collaboration, co-occurrence, or co-citation relationships between two nodes, with the thickness of the links proportional to the number of publications co-authored by two researchers or the simultaneous occurrence of two keywords, Colors represent different clusters (16).

CiteSpace, created by Professor Chaomei Chen of Drexel University, serves as a tool for visually representing and analyzing literature (17). In this study, we utilized it to analyze keywords and references.

Additionally, the global distribution and thematic evolution of publications were analyzed using the R package “Bibliometrix”. National collaboration analysis was also performed using the online platform https://bibliometric.com/ (18).

We analyzed annual publications using Microsoft Office Excel 2019. The 2023 Impact Factor (IF) and JCR (Journal Citation Reports) categories of the journal were obtained from the Journal Citation Reports by Clarivate Analytics.

## Results

### General trend

A total of 514 papers were searched and analyzed, including 473 articles and 41 review articles. The annual publication figures from 2005 to 2023 are as follows: **Figure 1B**. These papers were written by 3,669 authors who are from 50 countries/regions, and 1000 institutes and published in 292 different journals from 2005 to 2023. We further conducted a detailed visual analysis of the relationships among countries, institutions, authors, journals, and keywords. Additionally, we found the journal’s categorization and impact factor on the Clarivate Analytics platform.

### Countries and institutions analysis

**Figure 2:**
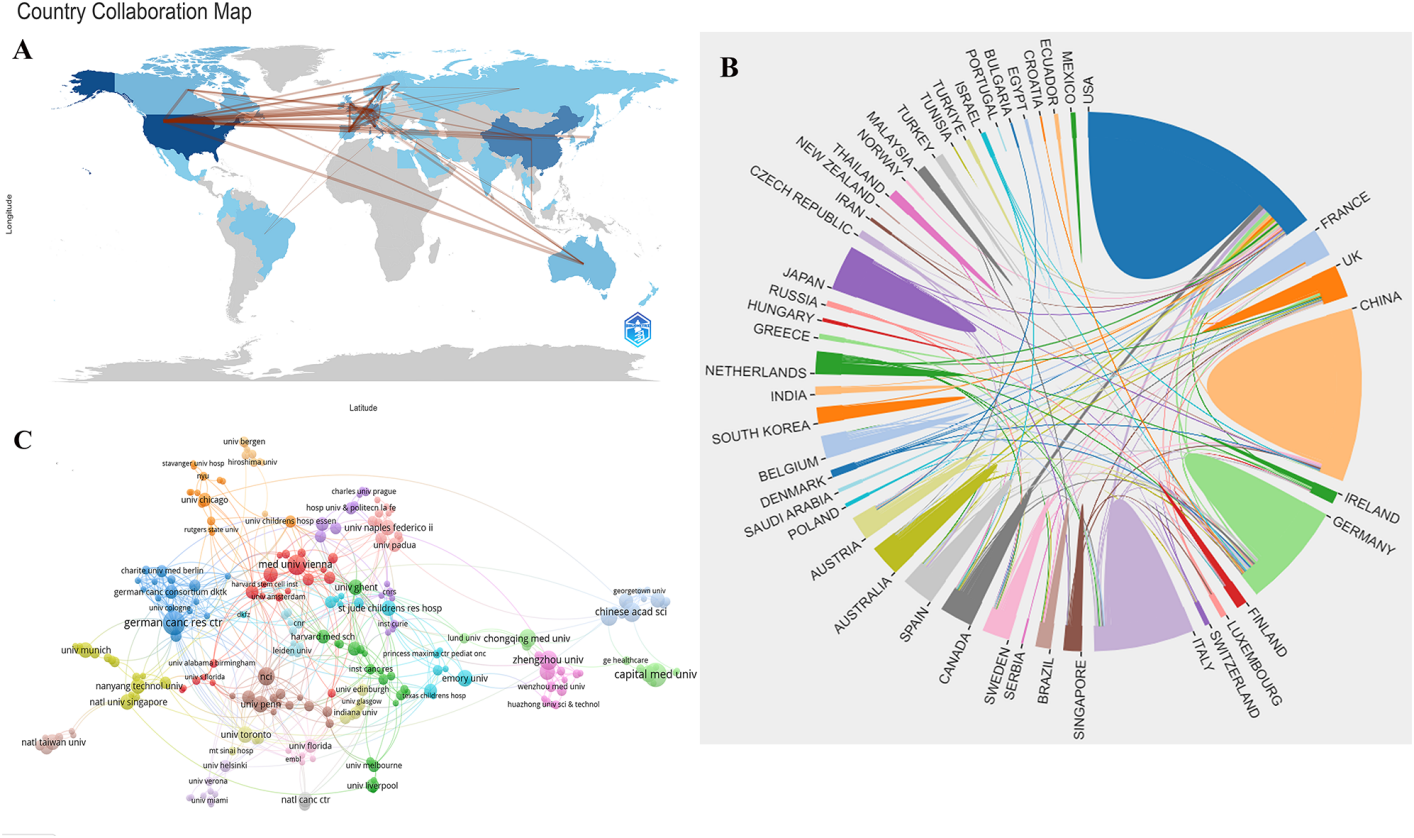
**(A)** Collaborative Map of Country in NB Omics Research. **(B)** Map of International Cooperation Between Countries/regions. **(C)** Map of visualization of institutions on research of omics in NB.

In the NB omics research, 50 countries and 1,000 institutions collaborated. The top 10 countries involved are distributed across North America, Asia, and Oceania. **Figure 2A** illustrates the map of international collaborations, with the United States being the most active collaborator, primarily with Germany, followed by Italy and the United Kingdom. **Table 1** shows the top 10 countries and institutions in the field of NB omics research. The United States (USA) leads in the number of publications (164, 31.9%), followed by China (96, 18.7%), Germany (67, 13.0%), and Italy (55, 10.7%). As depicted in **Figure 2B**, different-colored regions represent different countries/regions, with the size of each colored block indicating the number of published articles. The connecting lines between blocks signify collaboration between countries. Compared to other regions, countries represented by the blue, orange, and green blocks have a higher publication count. These blocks correspond to the United States, China, and Germany, respectively. The numerous connecting lines with the blue block indicate more frequent collaborations between the United States and other countries.

**Table 1:** Top 10 countries and institutions in the field of NB omics research.

Rank	Countries	Documents	Institution	Documents
1	America	164(31.9%)	Capital Medical University	14(2.7%)
2	China	96(18.7%)	German Cancer Research Center	14(2.7%)
3	Germany	67(13.0%)	Medical University of Vienna	11(2.1%)
4	Italy	55(10.7%)	Chinese Academy of Sciences	10(1.9%)
5	Japan	33(6.4%)	Zhengzhou University	10(1.9%)
6	England	31(6.0%)	Chongqing Medical University	9(1.8%)
7	Canada	28(5.4%)	National Cancer Institute	9(1.8%)
8	Spain	26(5.1%)	Shanghai Jiao Tong University	9(1.8%)
9	Australia	21(4.1%)	Emory University	8(1.6%)
10	Austria	20(3.9%)	Guangzhou Medical University	8(1.6%)

In NB omics research, the top five institutions, according to publication rankings, are Capital Medical University (14, 2.7%), German Cancer Research Center (14, 2.7%), Medical University of Vienna (11, 2.1%), Chinese Academy of Sciences (10, 1.9%), and Zhengzhou University (10, 1.9%). In **Figure 2C**, each node symbolizes an institution, with the circle’s size proportional to the institution’s publication count. Node centrality indicates its frequency in shortest paths across the network, reflecting influence and significance. The connections between nodes indicate the strength of associations, with more connections implying greater collaboration.

### Journals and co-cited-journal analysis

The articles related to NB omics were published in a total of 292 journals. **Table 2** shows the top 10 journals in terms of publication volume and total citations, along with their corresponding Impact Factors (IF, JCR 2023). The top 10 journals are all JCR Q1 and JCR Q2.

**Table 2:** Top 10 journals and co-cited journals in NB omics research.

Rank	Journal	Publications	JCR	IF	Co-cited journal	Citations	JCR	IF
1	Journal of Proteome Research	23	Q1	4.4	Journal of Biological Chemistry	828	Q2	4.8
2	PLoS ONE	11	Q2	3.7	Nature	815	Q1	64.8
3	Molecular and Cellular Proteomics	10	Q1	7	Proceedings of the National Academy of Sciences of the United States of America	748	Q1	11.1
4	Oncogene	9	Q1	8	Cancer Research	618	Q1	11.2
5	Scientific Reports	9	Q2	4.6	PLoS ONE	493	Q2	3.7
6	Cancers	8	Q1	5.2	Journal of Clinical Oncology	487	Q1	45.3
7	Journal of Biological Chemistry	8	Q2	4.8	Cell	460	Q1	64.5
8	Oncotarget	8	Q2	4	Science	441	Q1	56.9
9	Pediatric Blood & Cancer	8	Q1	3.2	Oncogene	410	Q1	8
10	International Journal of Molecular Sciences	7	Q1	5.6	Nucleic Acids Research	377	Q1	14.9

The journal boasting the most publications is the Journal of Proteome Research (n=23, 4.5%), followed by PLoS ONE (n=11, 2.1%), and Molecular and Cellular Proteomics (n=10, 1.9%).

**Figure 3:**
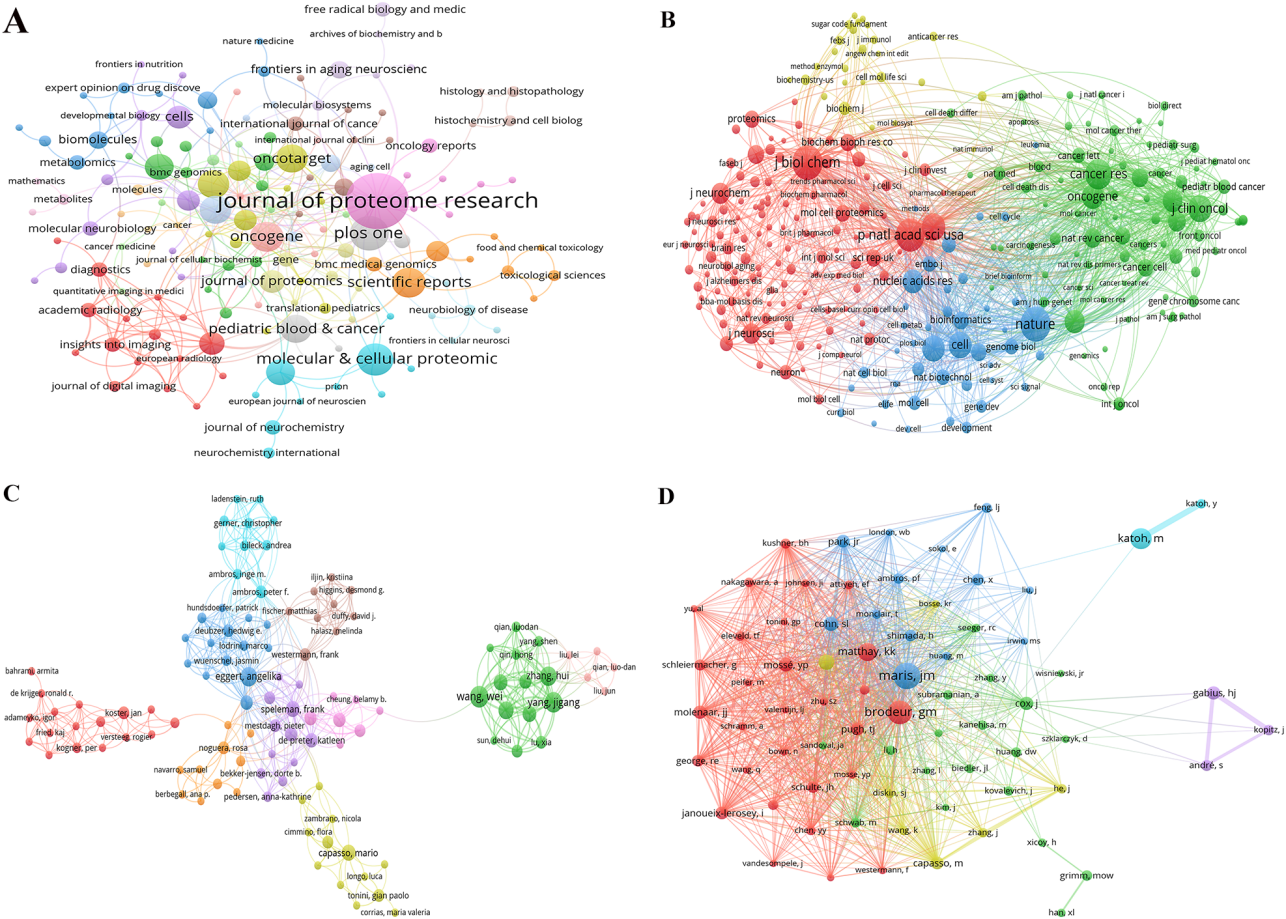
**(A)** Network of journals on research of omics in NB. **(B)** Network of co-cited journals on research of omics in NB. **(C)** Network of authors on research of omics in NB. **(D)** Network of co-cited authors on research of omics in NB.

**Figure 3A** shows the network diagram of NB omics related journals, with each node represents a journal, with nodes of the same color indicating journals that are frequently cited together within the network. The size of each node reflects the number of papers published by the journal, while the thickness of the links indicates the strength of the citation relationship between journals, representing the frequency with which one journal cites another. Co-citation frequency refers to the rate at which two documents are cited in conjunction. In the 3,254 journals collectively cited, the Journal of Biological Chemistry had the highest co-citation frequency (n=828), followed by Nature (n=815), and Proceedings of the National Academy of Sciences of the United States of America (n=748). Furthermore, Nature had the highest impact factor (IF=64.8), followed by Cell (IF=64.5). In **Figure 3B**, we present the co-citation network of journals with a frequency equal to or exceeding 20. Notably, PLoS ONE stands out as the sole journal listed in the top 10 for both publication and citation counts.

### Authors and co-cited authors analysis

The NB omics study involved a total of 3,669 authors. **Table 3** presents the top 10 authors in this field. Wang, W emerged as the most prolific scholar with 10 published articles, followed closely by Yang, JG, who contributed 9 articles. **Figure 3C** shows the collaboration network among authors. The different color clusters represent various research themes among author groups, with authors within the same color cluster typically collaborating on similar topics. The research directions for different groups are as follows: red for proteomics, blue for gene expression, cyan for growth, orange for identification, purple for cells, light purple for differentiation, brown for apoptosis, light yellow for genomics, green for classification, and light pink for prognosis. Among the authors, Wang, W., Yang, JG, and Eggert, A. stand out due to the large number of papers they have published, resulting in larger nodes. This indicates that Wang, W. and Yang, JG demonstrate close collaboration on the topic of NB classification. The most frequently cited authors are Maris, JM (n=139), followed by Brodeur, GM (n=116), and Katoh, M (n=95). **Figure 3D** depicts a network graph of co-cited authors with citations equal to or greater than 15. Nodes of different colors represent authors with distinct collaboration relationships, while nodes of the same color indicate authors within the same cluster. The size of nodes and the thickness of connecting lines positively correlate with the frequency of co-citations.

**Table 3:** The top 10 authors and co-cited authors on the research of omics in NB.

Rank	Author	Documents	citations	Co-cited authors	citations
1	Wang, W	10	233	Maris, JM	139
2	Yang, JG	9	55	Brodeur, GM	116
3	Eggert, A	8	189	Katoh, M	95
4	Zhang, H	8	48	Matthay, KK	72
5	Feng, LJ	7	40	Cheung, NKV	56
6	Gabius, HJ	7	560	Cohn, SL	48
7	He, J	7	49	Mossé, YP	47
8	Andre, S	6	502	Cox, J	44
9	Capasso, M	6	66	Gabius, HJ	44
10	Kan, Y	6	30	Molenaar, JJ	42

### Analysis of keywords and frontiers

**Table 4** displays the top 20 most frequently occurring keywords in the NB group omics. Two keywords, Neuroblastoma (n=174) and Expression (n=112), have frequencies exceeding 100 times each. The total link strength of seven keywords surpasses 200, while the remaining keywords exceed 100.

**Table 4:** The top 20 keywords with the highest occurrence of NB omics and their total link strength.

Rank	keyword	Occurrences	Total link strength	Rank	Occurrences	Occurrences	Total link strength
1	Neuroblastoma	174	867	11	Alzheimer’s Disease	32	141
2	Expression	112	597	12	Protein	30	149
3	Proteomics	89	453	13	N-Myc	29	160
4	Cancer	82	429	14	Survival	28	151
5	Genomics	51	281	15	Activation	27	122
6	Identification	50	241	16	Differentiation	26	144
7	Oxidative Stress	49	268	17	Metabolism	26	150
8	Apoptosis	39	198	18	Neuroblastoma Cells	24	113
9	Cells	37	194	19	Biology	23	121
10	Gene-Expression	34	163	20	Brain	23	111

**Figure 4:**
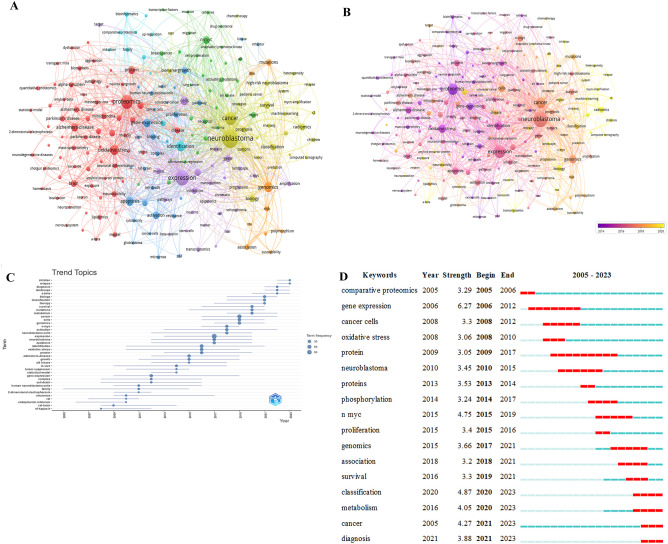
**(A)** Network of keyword on research of omics in NB. **(B)** Keyword co-occurrence network analysis diagram on research of omics in NB. **(C)** Trending topics on research of omics in NB. **(D)** The top 17 keywords with the strongest citations on research of omics in NB.

**Figure 4A** displays keywords with an occurrence equal to or greater than 5 times, resulting in a total of 7 clusters representing 7 research directions. The closest 5 keywords in each of these 7 major clusters (red, green, blue, yellow, purple, cyan, orange) are as follows: Red: proteomics, oxidative stress, Alzheimer’s disease, metabolism, protein; Green: n-myc, mycn, receptor, breast-cancer, activating mutations; Blue: gene expression, apoptosis, activation, pathway, binding; Yellow: neuroblastoma, cancer, classification, radiomics, biology; Purple: expression, cells, differentiation, gene, inhibition; Cyan: identification, phosphorylation, bioinformatics, mass spectrometry, comparative proteomics; Orange: genomics, mutations, risk, association, polymorphism.

**Figure 4B** displays the clustering analysis graph based on keywords, with different colors indicating popular keywords from different years. Lighter colors represent more recent publication years.

**Figure 4C** illustrates the trend analysis of NB omics themes to further insight into the trends in the field. The keywords from 2008 to 2011 include NF-kappa-B, rat brain, endoplasmic reticulum, rat, micrornas, 2-dimensional electrophoresis, family, human neuroblastoma cells. The keywords from 2021 to the present are: survival, therapy, classification, biology, a-beta, landscape, diagnosis, relapse, children.

**Figure 4D** lists the top 17 keywords with the most significant citation bursts, sorted by the starting year. Blue bars represent published references, while red bars indicate citation bursts. The earliest is “comparative proteomics,” followed by “gene expression,” “cancer cells,” and “oxidative stress.” In recent years, the key terms include “classification,” “Metabolism,” “Cancer,” and “Diagnosis”.

### Analysis of references

The co-cited references for NB omics amount to a total of 25,042 articles, with 26 of them being cited more than 20 times. **Table 5** illustrates the top 10 cited references, among which 2 articles have been cited 50 times or more, while the rest have citations exceeding 20 times.

**Table 5:** The top 10 co-cited references on the research of omics in NB.

Rank	Article Title	Author	Journal	Year	Citations	DOI
1	Neuroblastoma	John M Maris	Lancet	2007	55	10.1016/s0140-6736(07)60983-0
2	Recent advances in neuroblastoma	John M Maris	New England Journal of Medicine	2010	50	10.1056/nejmra0804577
3	The International Neuroblastoma Risk Group (INRG) classification system: an INRG Task Force report	Susan L Cohn	Journal of Clinical Oncology	2009	47	10.1200/jco.2008.16.6785
4	Neuroblastoma: biological insights into a clinical enigma	Garrett M Brodeur	Nature Reviews Cancer	2003	46	10.1038/nrc1014
5	The genetic landscape of high-risk neuroblastoma	Trevor J Pugh	Nature Genetics	2013	39	10.1038/ng.2529
6	Neuroblastoma	Katherine K Matthay	Nature Reviews Disease Primers	2016	34	10.1038/nrdp.2016.78
7	Identification of ALK as a major familial neuroblastoma predisposition gene	Yaël P Mossé	Nature	2008	31	10.1038/nature07261
8	Neuroblastoma: developmental biology, cancer genomics and immunotherapy	Nai-Kong V Cheung	Nature Reviews Cancer	2013	30	10.1038/nrc3526
9	Somatic and germline activating mutations of the ALK kinase receptor in neuroblastoma	Isabelle Janoueix-Lerosey	Nature	2008	26	10.1038/nature07398
10	Activating mutations in ALK provide a therapeutic target in neuroblastoma	Rani E George	Nature	2008	25	10.1038/nature07397

**Figure 5:**
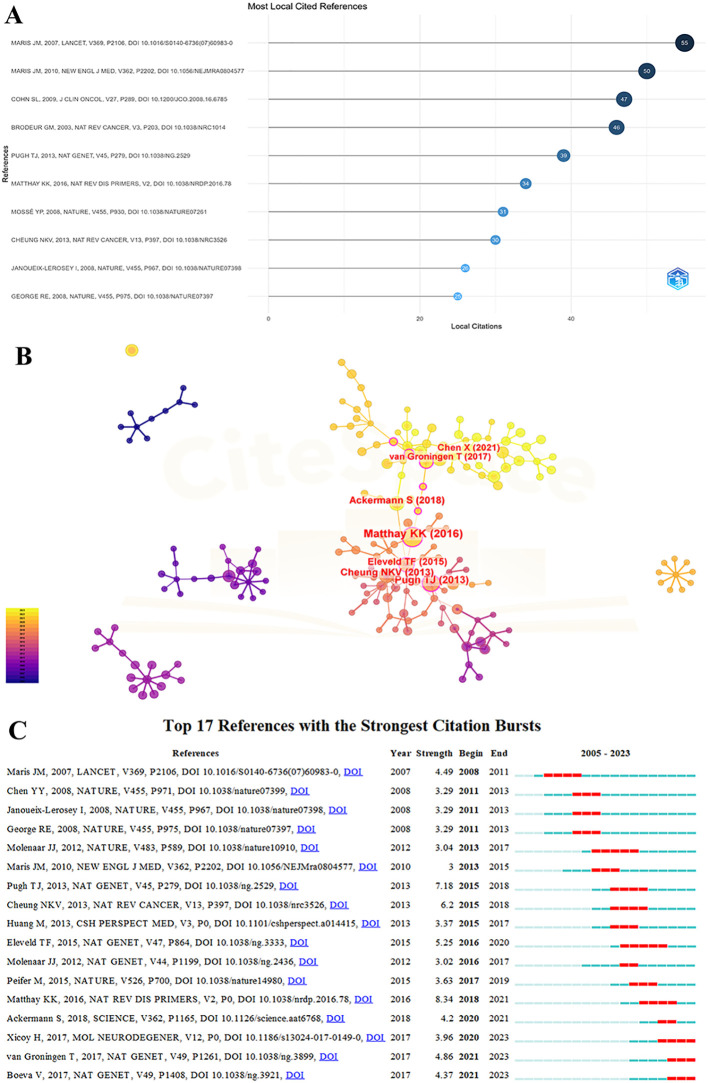
**(A)** MOST local cited referecnces on the research of omics in NB. **(B)** reference’ map on the research of omics in NB. **(C)** The top 17 references with the strongest citation bursts for publications on omics in NB.

**Figure 5A** displays the MOST locally cited references in NB omics. Specifically, these are the references with the highest citation counts within the selected literature set about NB omics that we studied. The most frequently cited reference is a paper by John M. Maris, published in Lancet in 2007, titled “Neuroblastoma,” which has been cited 55 times. The second most cited reference is also by John M. Maris, published in the New England Journal of Medicine in 2010, titled “Recent advances in neuroblastoma,” with 50 citations. **Figure 5B** presents references in the field of NB omics, with the highest number of citations going to the paper by Katherine K. Matthay published in 2016, titled “Neuroblastoma” (n=20), followed by Trevor J. Pugh’s 2013 paper titled “The genetic landscape of high-risk neuroblastoma” (n=15).

**Figure 5C** depicts the top 17 references experiencing the most substantial citation surges in NB omics. The references are arranged by the year of origin, with blue bars representing time intervals and red bars indicating the start and end years of each citation surge. These surges first appeared in 2008, with an intensity range from 3.0 to 8.34. The reference with the highest citation surge intensity (8.34) was a paper published in 2016 in Nature Reviews Disease Primers, titled “Neuroblastoma.” The second-highest intensity citation surge (7.18) was associated with a paper published in 2013 in Nature Genetics, titled “The genetic landscape of high-risk neuroblastoma”.

## Discussion

### General information

In this research, a bibliometric analysis of 514 articles in NB omics from the WOSCC database was carried out using VOSviewer, CiteSpace, and Bibliometrix. The objective was to visually analyze research hotspots and trends in the NB omics field. Findings indicated a consistent growth in publications since 2005, averaging 8.3 articles annually from 2005 to 2020. A notable increase in relevant papers was observed from 2015 to 2020, indicating an outbreak period in NB omics research during that time. After 2020, there was a rapid surge in the number of relevant papers, suggesting a growing interest among scholars and the gradual entry of NB omics into a mature development stage.

**Table 1** highlights the prominent contributors to this field, namely the United States, China, and Germany. The United States leads in both publication numbers and collaborations with other countries or regions. Six of the top ten institutions, based on the number of published papers, hail from China. The institution boasting the most publications is the Capital Medical University in China, followed by the German Cancer Research Center in Germany. While China ranks second in the number of publications, Chinese institutions lack collaboration with other countries or regions, and there is a need for active cooperation with other institutions to promote the development of Chinese NB omics.

The journals and co-cited counterparts fall into three main categories, namely biological and molecular journals, comprehensive journals, and oncology journals, all positioned within the Q1 or Q2 categories. The Journal of Proteome Research (IF = 4.4, Q1) boasts the highest publication count among scientific journals in the field, followed by PLoS ONE (IF = 3.7, Q2), and the Journal of Biological Chemistry (IF = 4.8, Q2) has the highest number of co-citations.

Wang, W from the United States stands out as the most prolific author, boasting the highest publication count among researchers in the field. In 2021, Wang published an article titled “Molecular targeting therapies for neuroblastoma: Progress and challenges,” summarizing research on molecular pathways, such as MYCN, BIRC5, PHOX2B, LIN28B, and epigenetic regulatory factors, in the current understanding of the mechanisms underlying NB development (19). This reflects the ongoing focus on molecular mechanisms in NB research, which is consistent with the discussion of current research trends and hotspots. In the realm of co-cited authors, the most frequently cited individual is Maris, JM. His most cited article, published in 2017 under the title “Advances in the translational genomics of neuroblastoma: From improving risk stratification and revealing novel biology to identifying actionable genomic alterations,” summarizes the knowledge in NB genetics and genomics. The article emphasizes that a deeper understanding of lineage susceptibility, recurrent segmental chromosomal changes, somatic point mutations, translocations, and the latest research on the clonal evolution of recurrent NB can offer new possibilities for improving prognosis and exploring potential therapeutic opportunities (20).

The second most frequently cited author is Brodeur, GM, whose article titled “Neuroblastoma: developmental biology, cancer genomics, and immunotherapy” was published in 2013. This publication offers a refreshed insight into the NB neural crest and its cellular origins, emphasizing the unique potential therapeutic targets in NB. The article also analyzed the efficacy of anti-ganglioside GD2 antibody therapy in immunosuppressive tumor microenvironments and proposed future translational research roadmaps, including high-throughput drug screening and next-generation animal models (21). It emphasized the key focus on treatment targets for NB and future research directions.

### Hotspots and frontiers

Exploring hot keywords, citation burst references, and trending topics aids in grasping the current research trends and frontiers within this domain (22). By analyzing through bibliometrics and visualization software, we summarize the hot topics in the academic field, objectively evaluate the forefront research directions in the field of NB omics. The current research focus includes the identification of potential biomarkers using omics in NB and the application of personalized medicine, as well as the challenges and future directions in NB omics.

#### Applications of omics in the identification of potential biomarkers and personalized medicine in NB

Our analysis of hot keywords, citation bursts, and trending topics reveals current research focuses on identifying biomarkers and personalized medicine in NB. The results highlight key genetic mutations and susceptibility loci, such as PHOX2B and ALK, which are central to familial and sporadic NB.

NB is categorized into familial NB and sporadic NB, with familial NB accounting for 2% of all NB cases (23). The first susceptibility gene mutation identified by NB is in PHOX2B, which contains two polyalanine repeat sequences. This gene encodes a paired-like homeobox transcription factor with functions that can promote cell cycle exit and neuronal differentiation (24, 25). The most common genetic mutation linked to familial NB occurs in the ALK receptor tyrosine kinase gene expressed in the sympathetic adrenal lineage during neural crest development (23, 26–28). This gene is implicated in modulating the equilibrium between proliferation and differentiation via diverse cellular pathways, including the mitogen-activated protein kinase (MAPK) and Ras-related protein 1 (RAP1) signaling pathways (29, 30). In addition, PHOX2B can directly regulate the expression of the ALK gene (31). The susceptibility single nucleotide polymorphisms (SNPs) associated with familial NB in genome-wide association studies (GWAS) include: BARD1, DUSP, DDX4-IL31RA, HACE1, HSD17B, LMO1, LIN28B, LINC00340, and LOC729177 (FLJ44180) (32–35).

In sporadic NB, PHOX2B mutations and GWAS susceptibility loci are infrequent. Activating mutations in ALK are found in about 6-10% of patients, while high-level ALK gene amplification is present in 3-4% of cases (23, 26, 36). The most common genetic mutation in sporadic NB involves the amplification of MYCN, expressed in the neural crest during development and plays a crucial role in regulating the proliferation, growth, differentiation, and survival of cells within the developing central nervous system. This amplification is observed in around 22% of patients and is linked to an unfavorable prognosis (37). Another common genetic mutation in sporadic NB is the ATRX mutation, and its relationship with the age of diagnosis is statistically significant. In NB patients harboring ATRX mutations, 17% manifest in children aged 18 months to 12 years with stage 4 disease, while 44% are observed in patients aged 12 and above (38).

Gene amplification in cancer and chromosomal aberrations may result in the dysregulation of messenger RNA, microRNA, and other non-coding RNAs, thereby disrupting cell apoptosis, differentiation, and immune surveillance (39, 40). The adverse prognosis in NB patients is specifically marked by deletions of 1p, 11q, and amplification of 17q. Additionally, deletions of 3p, 4p, 9p, and 14q, as well as amplifications of 1q, 2p, 7q, and 11p, also impact the prognosis (41, 42). The patient’s age, disease stage at the time of diagnosis, presence of MYCN amplification in NB cells, deletion of the 11q chromosome, histological characteristics, and ploidy have become crucial factors for stratifying risk groups in NB patients (43). High-risk NB patients have chromosomal rearrangements, and the frequency of these aberrations rises with the age at diagnosis, exerting a strong predictive impact on outcomes (44, 45).

Like genomics, proteomics research plays a crucial role in understanding the mechanisms of tumor formation in NB, discovering biomarkers, and elucidating pathways related to treatment response and drug resistance. Hsu’s study revealed a significant upregulation of GRP75, heat shock protein 2, protein disulfide isomerase A3 precursor, TCP1-containing chaperonin protein subunit 1β, and Eno1 protein during NB differentiation induction (46). GRP75, a member of the heat shock protein 70 family, is thought to function in tumor development control. Additionally, GRP78, another family member, together with GRP75, acts as a positive prognostic marker for NB in a separate study (47, 48). The mechanism involves the formation of the GRP75/78-RHAMM complex, which binds to hyaluronic acid-mediated motility receptor (RHAMM) associated with the progression and metastasis of cancer, and interacts with microtubules to stabilize them during the interphase, preventing microtubule depolymerization and promoting the progression of mitosis (49, 50).

Valérie employed surface-enhanced laser desorption/ionization time-of-flight mass spectrometry (SELDI-TOF-MS) to detect serum amyloid A (SAA) protein, revealing its close association with the prognosis of NB patients (51, 52). Targeted therapy for tumor-specific mutations can effectively and precisely eradicate cancer while sparing patients from the acute and chronic toxicities that may occur during chemotherapy and radiation therapy. However, in NB, traditional immunotherapy models are not applicable. Treatment approaches based on antibodies rather than T cells, especially those targeting cancer embryonic differentiation antigens, offer viable alternative strategies (21). Currently, research has found that the construction of T-cell vaccines targeting cytotoxic T lymphocytes (CTL) recognition, including cancer testis antigens (MAGE and NY-ESO-1), MYCN 110, and surviving in NB antigens, can stimulate T cell-mediated immunity to some extent (53–55).

GD2, a cancer embryonic differentiation antigen, is expressed not only during fetal development, but also in mature neurons, pain fibers, and skin cells (56). Treatment with anti-GD2 monoclonal antibodies (MAb) administered intravenously, particularly the anti-GD2 IgG antibodies (ch14.18 and murine 3F8), especially in combination with interleukin-2 (IL-2), granulocyte-macrophage colony-stimulating factor (GM-CSF), and oral 13-cis-retinoic acid (CRA), can effectively alleviate symptoms in high-risk NB patients. The potential mediating mechanism is granulocyte-mediated Antibody-Dependent Cellular Cytotoxicity (ADCC) and NK-ADCC (57, 58). Due to the depletion of white blood cells required for ADCC after induction chemotherapy or autologous stem cell transplantation, the combination of cytokines IL-2 and GM-CSF can be used with monoclonal antibodies (MAb). IL-2 can activate NK cells, natural killer T (NKT) cells, T cells, and regulatory T cells (T reg). Similarly, IL-15, like IL-2, can activate NK, NKT, and CD8+ T cells. However, in non-human primates studies, IL-15 does not lead to capillary leakage, activation-induced cell death, or an increase in T reg activation (59).

Proteomics research emphasizes the importance of biomarkers like GRP75 and GRP78 in NB. This is corroborated by Hsu’s research on these proteins and their roles in tumor development and prognosis. Our discussion on targeted therapies and antibody treatments aligns with current research trends and highlights advancements in personalized treatments for NB, such as the development of T-cell vaccines and anti-GD2 monoclonal antibodies. Zhang combined metabolomics and transcriptomics analyses, uncovering noteworthy distinctions in the cAMP, PI3K-Akt, and TNF signaling pathways between high-grade NB (HG-NB) and low-grade NB (LG-NB). HG-NB corresponds to stage 4 according to the International NB Staging System (INSS), while LG-NB includes stages 1, 2, and 3. Additionally, three biomarkers, MGST1, SERPINE1, and REBB3, were identified (60). The specific mechanism centers around cAMP as a second messenger, engaging in diverse cellular processes like growth, differentiation, and gene transcription. It influences four effector proteins, namely cAMP-activated exchange proteins, cyclic nucleotide-gated ion channels, myosin heavy chain proteins, and the cAMP-dependent PKA pathway (61, 62). The PI3K-Akt pathway exerts its influence on tumors through the stimulation of receptor tyrosine kinases and somatic mutations in pathway-specific components in somatic cells. Inhibiting this pathway can halt the progression of cancer (63). The dysregulation of PI3K-Akt in HG-NB has been linked to PTEN tumor suppressor gene dysfunction in several studies (64). In addition, a study found that activation of the PI3K-Akt signaling pathway can prompt cell death by suppressing autophagy (65). TNF plays a role in both tumor angiogenesis and cell death, promoting the progression and metastasis of tumors (66). Moreover, TNF can stimulate the expression and activation of downstream molecules, such as nuclear factor κB and p38 mitogen-activated protein kinase, thereby controlling diverse biological processes, including cell death (67).

The analysis conducted by Prasinou using fatty acid methyl ester (FAME) gas chromatography revealed that in apolipoprotein E (apoE), both apoE3 and apoE4 increased the levels of saturated fatty acids (SFA) and monounsaturated fatty acids (MUFA), as well as the omega-6/omega-3 ratio, in NBSK-N-SH cell membranes. They reduced total polyunsaturated fatty acids (PUFA), leading to decreased membrane stability indicators like PUFA balance, unsaturation index, and peroxidation index. Meanwhile, ApoE3 elevated stearic acid and dihomo-γ-linolenic acid (DGLA) levels, with apoE4 exhibiting the opposite impact. This study emphasizes the importance of membrane lipidomics in understanding the role of apolipoproteins in neurodegenerative diseases (68).

The integration of metabolomics and transcriptomics, as discussed by Zhang, emphasizes the importance of pathway analyses in distinguishing between high-grade and low-grade NB. This reflects our results on the differential signaling pathways and biomarkers associated with various NB grades. Radiomics, a swiftly advancing domain, concentrates on the extraction and analysis of numerous quantitative features from medical images. Utilizing advanced image processing algorithms, radiomics transforms medical images into rapidly exploitable data (69). These characteristics encompass information about tumor morphology, voxel texture, intensity, and spatial relationships within the tumor and its surrounding environment. In NB, various medical imaging modalities are currently employed, including computed tomography (CT), positron emission tomography-computed tomography (PET-CT), magnetic resonance imaging (MRI), and metaiodobenzylguanidine (MIBG) scintigraphy.

The International Neuroblastoma Pathology Classification (INPC) is an important classification system for NB. It categorizes NB into two subgroups, favorable histology (FH) and unfavorable histology (UFH), based on several risk factors linked to poor prognosis such as patient age, tumor histologic subtypes, differentiation grade, and mitosis-karyorrhexis index (MKI). This classification system provides a reference for different treatment stratifications (70). The pathological heterogeneity of NB results in inconsistent outcomes when multiple pathologists analyze the same patient. The heterogeneity within tumors results in diverse levels of differentiation across different tumor regions, diminishing the accuracy of the INPC. Assessing the MKI proves challenging and subjective, primarily due to the requirement for manually counting 5000 cells under a microscope (71). However, radiomics can automate and objectively extract quantitative features to reflect the heterogeneity of lesions, providing significant advantages in tumor staging, pathological subtypes, and prognosis prediction (72).

Studies indicate that radiogenomics has the potential to identify the pathological subtypes and genetic aberrations of NB (73). WU has developed a radiomics model based on CT images to predict MYCN amplification in pediatric NB. In the training group, the model achieved an Area Under the Curve (AUC) of 0.93, compared to 0.92 in the testing group (74). Wang employed a CT-based radiomics method for predicting the INPC of NB. The AUC values in the training and testing groups were 0.851 and 0.816, respectively. Decision curve analysis further validated the radiomics model’s outstanding performance across various high-risk thresholds (75). Qian created a radiomics model utilizing 18F-FDG PET/CT images for distinguishing INPC subgroups. The model demonstrated AUCs of 0.877 and 0.868 in the training and validation cohorts, respectively (76).

#### Challenges and future directions in NB omics

Due to the smaller number of pediatric patients compared to adults and significant differences in drug metabolism, acute toxicity, and late effects between children and adults, the translation and clinical research of pediatric cancer face a series of challenges compared to adult cancer. Additionally, most preclinical studies of pediatric cancers use murine cell lines or xenograft models, but for immunological analysis, mouse effector cells are not ideal human surrogates. To address this issue, it is recommended to use humanized mice implanted with human immune cells to better simulate the human immune system. The high-throughput capabilities of proteomic technologies provide a rich source for discovering potential biomarkers. In the future, NB proteomics can explore biomarkers through the study of protein fragment subtypes, glycoproteins, phosphorylated proteins, and autoantibodies (77). In future experiments using mouse models, it is possible to test the rational integration of antitumor monoclonal antibodies, effector cells, and cytokines for inducing tumor cell death. Additionally, the inclusion of small molecule drugs can be explored to prevent the escape of cell death (21).

The radiomic approach in NB holds the potential to enhance diagnostic accuracy, treatment planning, and prognosis prediction. However, realizing these goals faces several challenges. Firstly, the limited sample size of NB, due to its relative rarity, makes it difficult to obtain large sample cohorts with sufficient clinical and imaging data for radiomic analysis. Collaborative efforts, data sharing, and multi-institutional cooperation are essential means to overcome this limitation. Secondly, despite the quantitative nature of radiomics, transforming these features into biologically relevant information remains challenging. Lastly, the inherent heterogeneity in imaging protocols and data collection methods, stemming from multiple centers, may impact the reproducibility of research results. Implementing standardized imaging protocols, promoting the sharing of imaging datasets, and developing coordinated technologies are effective strategies to address this issue.

Another current issue is that most preclinical studies on childhood cancers, including NB, are short-term, utilize clinically irrelevant drug doses and regimens, fail to adequately consider combination chemotherapy, and lack appropriate benchmarks. Moreover, these studies often lack statistical design, proper randomization of animal groups for treatment plans, and mechanisms to keep the research team “blind” to the treatments administered to the study subjects. To address these issues, future efforts should involve building interdisciplinary teams comprising clinical researchers, laboratory scientists, pharmacologists, and biostatisticians.

The future direction of NB omics research should pivot towards interdisciplinary translational research teams that utilize validated preclinical models. These teams seek to pinpoint optimal combinations of molecularly targeted therapies, broad-spectrum chemotherapy, and immunotherapy to treat patients with unfavorable prognoses. Simultaneously, they aim to scale down treatment intensity for individuals with more favorable prognoses. The primary objective is to prioritize targeted therapies and minimize cytotoxic treatments, with the overarching goal of curing NB and mitigating treatment-related adverse effects in pediatric patients. To advance treatment strategies for NB, it is critical to delve deeper into the molecular mechanisms of NB tumorigenesis and progression, identifying key signaling pathways and molecular targets, and understanding the tumor microenvironment’s role in NB progression and metastasis. Developing novel therapeutic strategies, such as targeted therapies and immunotherapies, is essential to improve the prognosis for high-risk NB patients. Personalized medicine approaches that consider the genetic and molecular heterogeneity of NB tumors are also crucial. Addressing the challenges of tumor heterogeneity requires strategies to target diverse subpopulations of tumor cells within a single NB tumor, including combination therapies and single-cell omics approaches. Standardizing omics data processing and analytical methodologies is vital to ensure the reproducibility and reliability of research. This includes developing standardized protocols for sample collection, data processing, and analysis, as well as establishing large-scale, multi-center omics databases to validate findings and improve the reliability of NB omics research.

### Limitations

This study has certain limitations. Firstly, Our search was confined to the WoSCC, which may overlook influential articles from PubMed or Scopus, potentially impacting the final results. However, WoSCC, a citation index database encompassing around 34,000 core journals worldwide and spanning various disciplines, stands out as the most commonly used and most suitable database for bibliometric analysis (78).

Secondly, this study only includes English articles and reviews, thereby excluding some non-English publications. Additionally, the WoSCC database remains open and is updated daily with new research, which may result in the omission of the latest publications and incomplete descriptions. However, the collection and analysis for this study were conducted within a short time frame, and the research findings essentially align with the latest statistical data. Therefore, our study still offers relatively objective information and insights.

## Conclusion

Our bibliometric analysis of NB omics publications involved 514 articles, showcasing a significant surge since 2020. This trend indicates a growing interest in NB omics, suggesting its gradual entry into a mature development stage. The analyzed articles span 50 countries and involve 1,000 institutions, 3,669 authors, and 292 journals. The United States led in publications and collaborations, particularly with Germany. Capital Medical University and the German Cancer Research Center are the institutions with the most published papers. The Journal of Proteome Research and the Journal of Biological Chemistry were the most prolific in terms of publications and co-citations, respectively. Scholars Wang, W, and Maris, JM, were identified as the most prolific authors in terms of publication quantity and co-cited authors in this field. “Neuroblastoma” and “Expression” were the most frequently occurring keywords, while “classification,” “Metabolism,” “Cancer,” and “Diagnosis” emerged as recent keywords. An article titled “Neuroblastoma” by John M. Maris had the highest number of citations among the references. We explored the potential applications of omics in identifying biomarkers and personalized medicine for NB, as well as challenges and future directions in NB omics. In conclusion, our study unveils NB omics research trends and frontiers. This study empowers scholars with a more scientific, objective, and comprehensive understanding of the field, fostering its development.

## Data Availability

The original contributions presented in the study are included in the article/supplementary material. Further inquiries can be directed to the corresponding author/s.
